# Guava leaf extracts promote glucose metabolism in SHRSP.Z-Leprfa/Izm rats by improving insulin resistance in skeletal muscle

**DOI:** 10.1186/1472-6882-13-52

**Published:** 2013-03-01

**Authors:** Xiangyu Guo, Hisae Yoshitomi, Ming Gao, Lingling Qin, Ying Duan, Wen Sun, Tunhai Xu, Peifeng Xie, Jingxin Zhou, Liansha Huang, Tonghua Liu

**Affiliations:** 1 Department of Endocrinology, Dongfang Hospital of Beijing University of Chinese Medicine, No.6, 1st Area Fangxing Yuan, 100078, Fangzhuang Fengtai District, Beijing, China; 2 School of Pharmaceutical Sciences of Mukogawa Women’s University, 11-68 Koshien-Kyuban-cho, 663-8179, Nishinomiya, Hyogo, Japan; 3 Department of Chinese Medicine, Tongren Hospital of Capital Medical University, No.2, Chongwenmennei Street, 100730, Dongcheng District, Beijing, China; 4 Department of Science and Technology, Beijing University of Chinese Medicine, No.11, North 3rd-ring East Road, 100029, Chao Yang District, Beijing, China

## Abstract

**Background:**

Metabolic syndrome (MS) and type 2 diabetes mellitus (T2DM) have been associated with insulin-resistance; however, the effective therapies in improving insulin sensitivity are limited. This study is aimed at investigating the effect of Guava Leaf (GL) extracts on glucose tolerance and insulin resistance in SHRSP.Z-Leprfa/Izm rats (SHRSP/ZF), a model of spontaneously metabolic syndrome.

**Methods:**

Male rats at 7 weeks of age were administered with vehicle water or treated by gavage with 2 g/kg GL extracts daily for six weeks, and their body weights, water and food consumption, glucose tolerance, and insulin resistance were measured.

**Results:**

Compared with the controls, treatment with GL extracts did not modulate the amounts of water and food consumption, but significantly reduced the body weights at six weeks post treatment. Treatment with GL extracts did not alter the levels of fasting plasma glucose and insulin, but significantly reduced the levels of plasma glucose at 60 and 120 min post glucose challenge, also reduced the values of AUC and quantitative insulin sensitivity check index (QUICKI) at 42 days post treatment. Furthermore, treatment with GL extracts promoted IRS-1, AKT, PI3Kp85 expression, then IRS-1, AMKP, and AKT308, but not AKT473, phosphorylation, accompanied by increasing the ratios of membrane to total Glut 4 expression and adiponectin receptor 1 transcription in the skeletal muscles.

**Conclusions:**

These data indicated that GL extracts improved glucose metabolism and insulin sensitivity in the skeletal muscles of rats by modulating the insulin-related signaling.

## Background

The incidence of metabolic syndrome (MS) and type 2 diabetes mellitus (T2DM) is increasing worldwide [1,2]. MS and T2DM are mediated by insulin-resistance. Hence, therapeutic strategies for the intervention of MS and T2DM should center on the improvement of insulin sensitivity. Currently, the efficacy of available medicines in improving insulin sensitivity is limited. Therefore, the discovery of new medicines for the improvement of insulin sensitivity and understanding their therapeutic actions will be of great significance.

The skeletal muscles are important for glucose metabolism, particularly in individuals with insulin resistance [3]. Physiologically, insulin can bind to its receptor and lead to IR tyrosine kinase activation, which phosphorylates the downstream IR substrate family members, such as IRS-1. Subsequently, the activated IRS-1 can activate a cascade of phosphorylation-dephosphorylation reactions, including 85-kDa of phosphatidylinositol-3-kinase (PI3K-p85) and serine/threonine kinases Akt/PKB (e.g. Ser-473 and Thr-308 for the Akt/PKB isoform), leading to glucose transporter 4 (GLUT4) translocation and intracellular glucose metabolism [4]. Indeed, the multiple post-receptor intracellular defects, such as activation of the insulin-stimulated IRS/PI3K pathway, occur in the skeletal muscles of individuals with insulin resistance [5]. Therefore, the IRS/PI3K signal pathway in the skeletal muscles is a promising therapeutic target in the effective treatment of MS and T2DM.

Obesity is associated with the development of MS or T2DM [6] and can promote excess deposition of lipids in the muscle and liver, contributing to the development of insulin resistance. Adiponectin is an adipocyte-secreted adipokine [7] that can regulate insulin resistance, obesity, and T2DM [8]. Adiponectin, through its receptor, can activate the 5^′^-AMP-activated protein kinase (AMPK) in the skeletal muscles, which further stimulates the phosphorylation of acetyl coenzyme A carboxylase (ACC), fatty-acid oxidation, glucose uptake, and reduction of glucose levels. Therefore, up-regulation of adiponectin receptor expression and activation can activate the AMPK pathway and positively regulate glucose metabolism and insulin sensitivity in the skeletal muscles.

*Guava Leaf* (GL) has been widely used as a herbal medicine for diabetes patients in some countries [9,10]. GL extracts contain tannins, polyphenolic compounds, flavonoids, pentacyclic triterpenoids, guiajaverin, quercetin, and other chemical compounds, which could regulate hyperglycemia [11]. Indeed, GL extracts could inhibit the PTP1B activity and alpha-glucosidase enzymes to regulate glucose metabolism in db/db mice. Additionally, it could promote glucose uptake in hepatocytes and increase the activities of hepatic hexokinase and glucose-6-phosphate dehydrogenase in the liver of STZ-induced diabetic SD rats [12-15]. However, little is known about whether GL extracts can modulate glucose metabolism and insulin resistance in the skeletal muscles and their mechanisms.

SHRSP.Z-Leprfa/Izm (SHRSP/ZF) rats were derived by crossing the spontaneously hypertensive stroke-prone Izumo rats (SHRSP/Izm) with Zucker fatty rats to generate a genetic mutant in the fa locus of chromosome 5. The SHRSP/ZF rats spontaneously developed hyperglycemia, obesity, hyperleptinemia, hypertension, and other metabolic disorders, which resemble human MS [16]. This strain of rats has been used as an excellent model of MS for testing anti-diabetic natural products and determining their therapeutic mechanisms.

We hypothesized that GL extracts may play a role in improving insulin resistance via skeletal muscle, not just through the liver. Thus, in this study, we investigated the effects of oral treatment with the soluble GL ethanol extracts on glucose metabolism and insulin resistance in the skeletal muscles of SHRSP/ZF rats.

## Methods

### Plant materials

*Guava Leaf* (Folium Psidii Guajavae Psidium guajava L.) was purchased from Sichuan Medical Pharmaceutical, Chengdu, China, and identified botanically by Pro. Chunsheng Liu (School of Chinese Material Medica, Beijing University of Chinese Medicine, Beijing, China). A voucher specimen (No. 2009051603) was deposited in the Experimental Center of Beijing University of Chinese Medicine.

### Preparation of plant extraction

Ethanolic extracts were isolated using a modified previously reported method [17]. The air-dried powdered leaves of guava (5 kg) were extracted with 20 L of 70% (V/V) ethanol three times at room temperature for 2 h with reflux condenser. The extracts were combined and concentrated by a rotary evaporator (Büchi, Rotavapor R-215, Switzerland) to form a syrup under reduced pressure (45°C). Subsequently, the syrup was evaporated to dryness at 70°C in vacuo. Finally, the dried extract was grounded to pass through a 60-mesh filter, and approximately 0.92 kg powder was obtained.

### Animals

Male SHRSP/ZF rats at 6 weeks of age were obtained from Japan SLC (Shizuoka, Japan). All rats were housed in a specific pathogen-free facility with 22-24°C temperature, 40-60% humidity, 12-hour light and dark cycle, and free access to normal chow and water for one week at the School of Pharmaceutical Sciences of Mukogawa Women’s University. SHRSP/ZF rats were randomized and administered twice per day as follows: the experimental group with GL extracts (2 g/kg) by gavage for six weeks, which was made freshly and suspended in distilled water (10 ml) before each oral administration; and the control group with an equimolar solution of distilled water.

The rats used in the present study were maintained in accordance with guidelines of the care and use of animals in the field of physiological sciences established by the Physiological Society of Japan, and the study was approved by the ethics committee of Mukogawa Women’s University, which consisted of Prof. Semma M, Shinozuka K, Moriyama K, Miki T, Ikeda K, Nakamura K, Tanaka T, and Oku K.

### Measurement of fasting plasma glucose, oral glucose tolerance test, plasma insulin, triglycerides, cholesterol, and FFA

Rats were fasted overnight, and their fasting blood glucose levels were measured on day 0, 14, 28, and 42 post treatment using One-touch meter [18]. At the end of the experiment, blood samples were obtained from individual rats for the measurements of the concentrations of fasting plasma insulin, triglycerides, cholesterol, and free fatty acids (FFA).

Rats were fasted overnight and subjected to oral glucose tolerance test (OGTT) on day 28 and 42 post treatment. Briefly, after measuring fasting blood glucose levels, individual rats were administered orally with 2 g/kg BW of glucose and their blood glucose levels were measured at 30, 60, 90, and 120 min post glucose challenge. The area under the curve (AUC) over the levels of blood glucose in individual rats was calculated by the trapezoidal rule: {[15 × log(gluc0 min)] + [30 × log(gluc30 min)] + [45 × log(gluc60 min)] + [30 × log(gluc120 min)]/120} [19]. The quantitative insulin sensitivity check index (QUICKI) of individuals was calculated by (QUICKI = 1/[log(fasting insulin μU/ml) + log (fastin glucose mg/dl)]) [20].

### Biochemical assays

The concentrations of plasma insulin and FFA were assayed using specific ELISA kits (Wako Pure Chemical Industries, Osaka, Japan). The concentrations of plasma triglycerides and cholesterol were determined by regular biochemistry assays using specific kits (Wako Pure Chemical Industries) on an AU400 Biochemical Analyzer (Olympus, Japan).

### Western blot analysis

The skeletal muscle tissues were collected from individual rats and subjected to lysis. After quantification, the lysates were separated by SDS-PAGE and transferred onto PVF membranes. Subsequently, the membranes were blocked with 3% BSA and incubated with primary antibodies against IRS-1, PI3K-p85, Akt, AMPK-α, GLUT4, phosphorylated IRS-1, Akt 473, Akt 308, AMPK-α, and control β-actin (Cell Signaling Technology, Beverly, MA, USA). The bound antibodies were detected with HRP-conjugated second antibodies and visualized using the electrochemilluminescence kit (Amersham, UK). The relative levels of each target protein to control β-actin were determined by densitometry analysis using the Image J 6.0 (Media Cybernetics). Additional Western blot assays were performed for the characterization of membrane-associated GLUT4 on the purified muscle membrane samples.

### Quantitative RT-PCR

Total RNA was extracted from the skeletal muscle tissues using Sepasol-RNA I Super G (Nacalai Tesque, Kyoto) and reversely transcribed into cDNA using the ReverTra Ace qPCR RT Kit (Toyobo), according to the manufacturers’ instructions. The relative levels of adiponectin receptor-1 mRNA transcripts to control β-actin were determined by quantitative RT-PCR using THUNDERBIRD SYBR qPCR Mix and specific primers on the ABI Prism 7000. The sequences of primers were forward 5^′^TGAGGTACCAGCCAGATGTC3^′^ and reverse 5^′^CGTGTCCGCTTCTCTGTTAC3^′^ for adiponectin receptor-1 and forward 5^′^GGGAAATCGTGCGTGACATT3^′^ and reverse 5^′^GCGGCAGTGGCCATCTC3^′^ for β-actin. The data are expressed as the relative ratios of adiponectin receptor-1 to β-actin.

### Statistical analysis

Data are expressed as the mean ± S.D. The difference between groups was analyzed by Student’s t-test and ANOVA for repeated measures. A *P* value of less than 0.05 was considered statistically significant.

## Results

### GL extracts improve the glucose metabolism but do not affect insulin production in SHRSP/ZF rats

**Figure 1:**
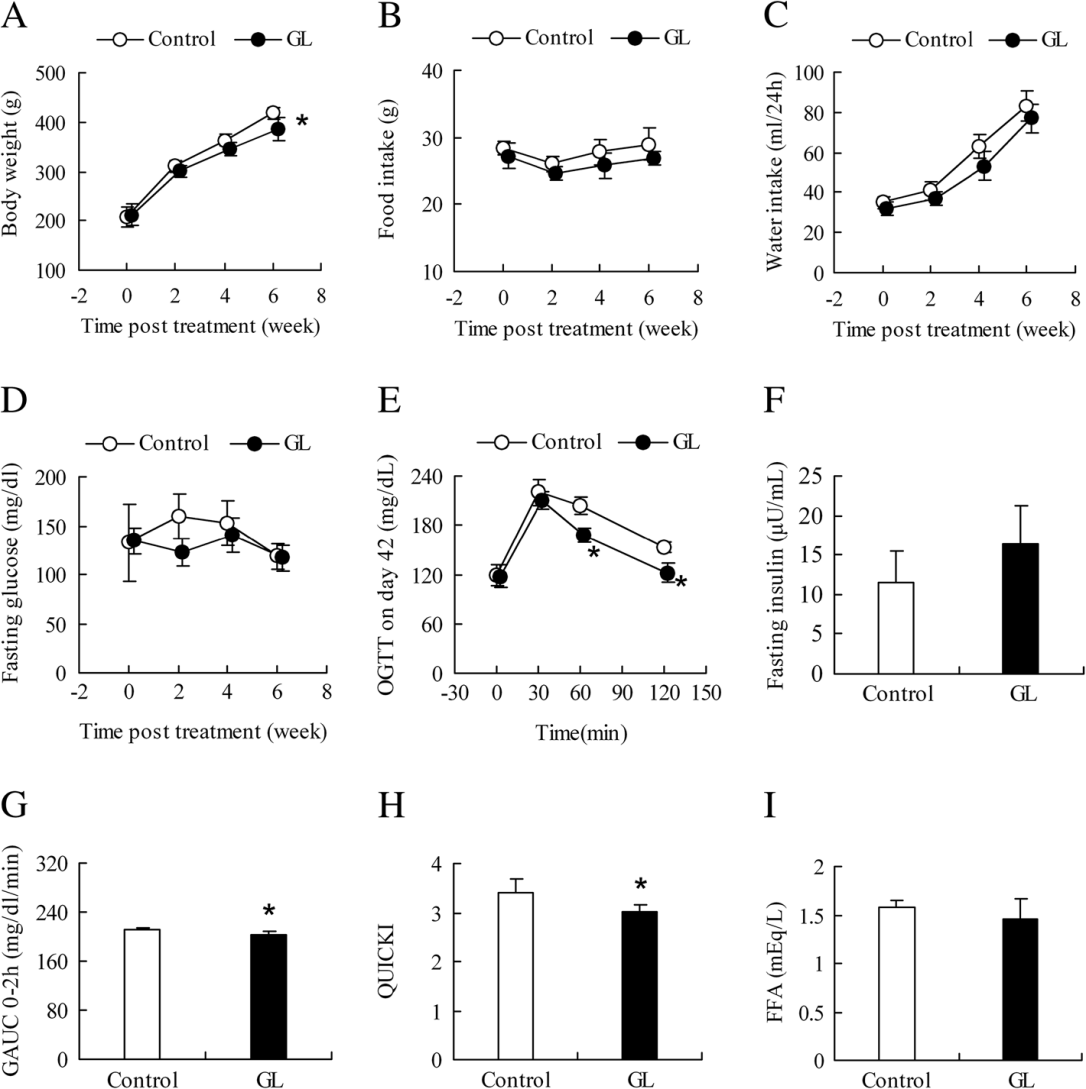
**The effects of GL extracts on the body weights and glucose tolerance in rats.** Male SHRSP/ZF rats at 7-weeks of age were administered with vehicle water or treated by gavage with 2 g/kg GL extracts daily for six weeks. Their body weights (**A**) and the amount of food (**B**) and water (**C**) consumption of individual rats were measured longitudinally before and after treatment. The levels of fasting blood glucose (**D**), oral glucose tolerance (**E**), fasting plasma insulin (**F**), GAUC0-2 (**G**), QUICKI (**H**), and FFA (**I**) at 6 weeks post treatment were measured. Data are expressed as the mean ± S.D. of individual groups of rats (n = 6 per group). * *p* < 0.05 vs. the control group.

To determine whether the GL extracts could affect glucose metabolism, SHRSP/ZF rats were fed with water as the controls or with GL extracts (2 g/kg) in water for six weeks. The body weights and the amount of food and water consumption of individual rats were measured in Figure 1. While the body weights in the controls increased with time, the body weights in the GL-treated rats at 2 and 4 weeks post treatment were slightly lower than that of the controls. The body weights in the GL-treated rats at 6 weeks post treatment were significantly lower than that of the controls (418.00 ± 12.98 vs. 385.00 ± 22.67 g, *p* = 0.01 n = 6) (Figure 1A). However, there was no significant difference in the amount of water and food consumption between the control and GL-treated rats (Figure 1B and C). Next, the levels of fasting blood glucose, oral glucose tolerance, and fasting plasma insulin in individual rats were tested longitudinally. There was no significant difference in the levels of fasting blood glucose between the controls and GL-treated rats before treatment and on day 14, 28 (data not shown), and 42 post treatment (Figure 1D). While there was no significant difference in the dynamics of blood glucose levels following oral glucose challenge on day 28 post treatment between the controls and GL-treated rats, the levels of blood glucose at 60 (204.18 ± 11.34 vs. 168.07 ± 9.15 mg/dl, *p* = 0.04, n = 6) and 120 minutes (153.62 ± 6.37vs. 121.83 ± 11.27 mg/dl, *p* = 0.03, n = 6) post glucose challenge on day 42 in the GL-treated rats were significantly lower than that in the controls (Figure 1E). As a result, the values of the AUC over the first 2-h after glucose challenge (GAUC_0-2_) in the GL-treated rats were significantly less than that of the controls (210.53 ± 5.15 vs. 203.08 ± 4.33 mg/dl/min, *p* = 0.02, n = 6, Figure 1G), and the QUICKI values in the GL-treated rats were significantly less than that in the control rats (3.40 ± 0.28 vs. 3.01 ± 0.12, *p* = 0.04, n = 6, Figure 1H). However, there was no significant difference in the levels of fasting plasma insulin (Figure 1F), FFA (Figure 1I), fasting plasma triglyceride, and cholesterol (data not shown) between the controls and GL-treated rats at 6 weeks post treatment.

### GL extracts enhance insulin sensitivity in the skeletal muscles by promoting the IRS-1-PI3K signal pathway in SHRSP/ZF rats

**Figure 2:**
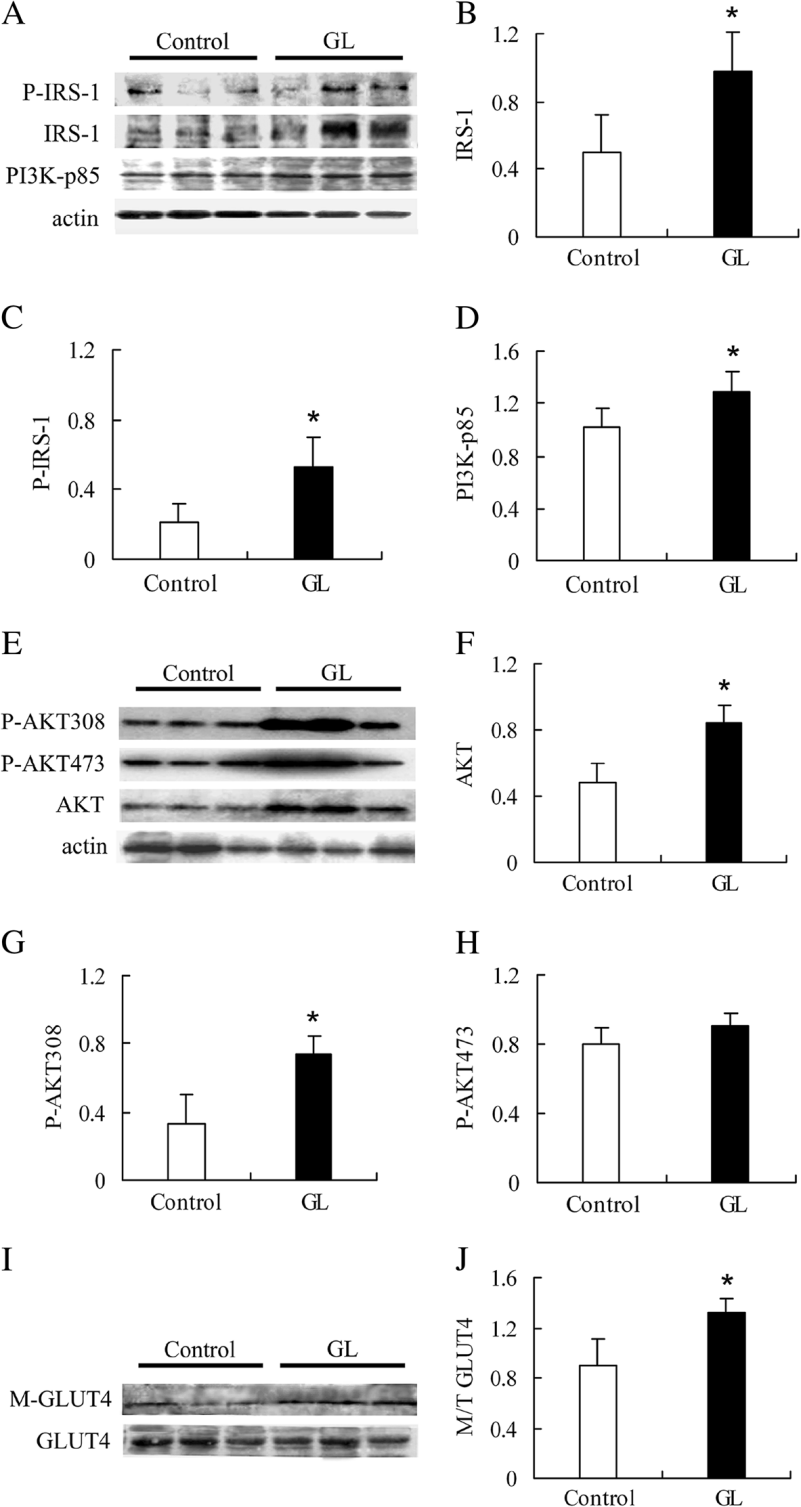
**The effects of GL extracts on skeletal muscles IRS-PI3k signaling in rats.** After the end of treatment, the levels of IRS-1 (**A** and **B**), phosphorylation IRS-1 (**A** and **C**), PI3K-p85 (**A** and **D**), Akt (**E** and **F**), phosphorylation Akt 308 (**E** and **G**), and Phosphorylation Akt 473 (**E** and **H**) in the skeletal muscles, as well as the membrane and total GLUT4 (**I** and **J**) in the skeletal muscles were characterized by Western blotting. Data are representative images or expressed as the mean ± S.D. of individual groups of rats (n = 4 per group). * *p* < 0.05 vs. the control group.

Insulin binds to its receptor, which phosphorylates the insulin receptor substrate (IRS)-1 and activates the PI3K pathway [21]. We tested whether GL extracts might improve insulin sensitivity in rats and characterized the levels of IRS-1 phosphorylation and PI3K in the skeletal muscles of different groups of rats by Western blot assays. The levels of IRS-1 (0.48 ± 0.25 vs. 0.98 ± 0.23, *p* = 0.02, n = 4, Figure 2A and B), phosphorylated IRS-1 (0.23 ± 0.11 vs. 0.54 ± 0.16, *p* =0.02, n = 4, Figure 2A and C), and PI3K-p85 (1.02 ± 0.13 vs. 1.29 ± 0.16, *p* = 0.03, n = 4, Figure 2A and D) in the muscles from the GL-treated rats were significantly higher than that in the controls. These data indicate that treatment with GL extracts significantly improved insulin sensitivity of the muscles in rats.

Activation of the PI3K pathway can further activate the protein kinase B/Akt pathway, which is a central intermediate for the metabolic actions of insulin [22]. We measured the relative levels of Akt, phosphorylated Akt 308, and Akt 473 in the muscles of rats, respectively. As shown in Figure 2, the relative levels of total Akt (0.44 ± 0.09 vs. 0.84 ± 0.11, *p* = 0.001, n = 4, Figure 2E and F) and phosphorylated Akt 308 (0.29 ± 0.17 vs. 0.74 ± 0.10, *p* = 0.005, n = 4, Figure 2E and G), but not Akt 473 (Figure 2E and H) in the muscles form the GL-treated rats were significantly higher than that in the controls. Finally, we found that similar levels of total GLUT4 were detected in the muscles from both groups of rats and that the levels of membrane-associated GLUT4 in the muscles from the GL-treated rats were obviously higher than that in the controls. As a result, the relative ratios of membrane-associated GLUT4 to total GLUT4 in the muscles form the GL-treated rats were significantly greater than that in the controls (0.89 ± 0.21 vs. 1.32 ± 0.17, *p* = 0.03, n = 4, Figure 2I and J). Therefore, treatment with GL extracts enhanced insulin-mediated GLUT4 translocation on the plasma membrane of muscle cells, leading to the improvement of insulin sensitivity.

### GL extracts improve the adiponectin receptor-1 gene expression in the skeletal muscles

**Figure 3:**
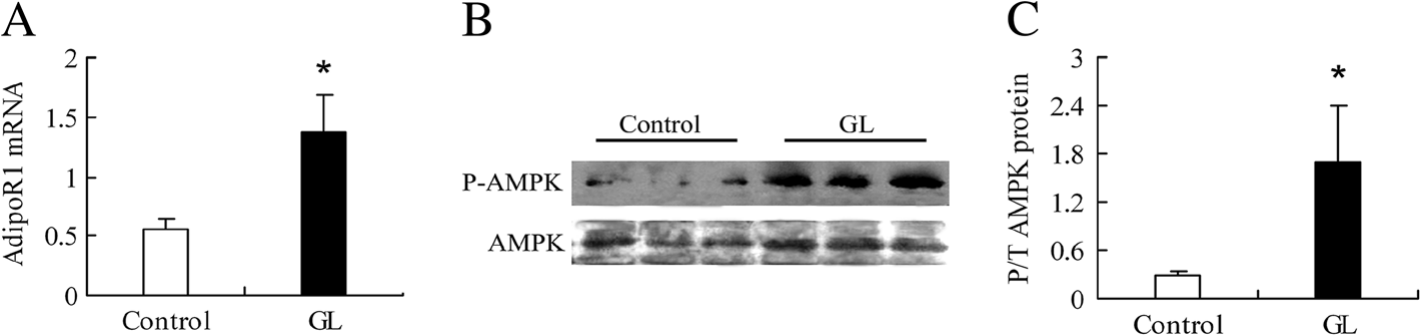
**The effect of GL extracts on skeletal muscles AdipoR1 expression and AMPK phosphorylation in rats.** The relative levels of AdipoR1 mRNA transcripts were determined by RT-PCR, and the levels of total AMKP expression and phosphorylation in the muscles of individual rats were characterized by Western blot assays. Data are representative images or expressed as the mean ± S.D. of individual groups of rats (n = 4 per group). (**A**) RT-PCR analysis of the AdipoR1 mRNA transcripts and (**B** and **C**) Western blot analysis of AMKP expression and phosphorylation. * *p* < 0.05 vs. the control group.

Low levels of plasma adiponectin are associated with the development of obesity and T2DM as well as hyperlipideamia [23]. Furthermore, elevated levels of triglyceride and FFA can interfere with insulin sensitivity in the muscles [24]. To further elucidate the mechanisms underlying the action of GL extracts, we characterized the relative levels of adiponectin receptor mRNA transcripts in the skeletal muscles from different groups of rats by quantitative RT-PCR. We found that the relative levels of adiponectin receptor-1 (AdipoR1) mRNA transcripts in the muscles from the GL-treated rats were significantly higher than that in the controls (0.56 ± 0.07 vs. 1.37 ± 0.31, *p* = 0.02, n = 4, Figure 3A).

### GL extracts improve the AMPK phosphorylation in the skeletal muscles

Dysregulation of fatty acid metabolism plays a pivotal role in the pathogenesis of insulin resistance in the skeletal muscle [25]. Adiponectin can activate the AMPK, which is crucial for glucose and lipid metabolism in the skeletal muscles. Finally, we examined the levels of AMPK activation in the muscles of different groups of rats and found that the relative levels of phosphorylated AMPK to total AMPK in the muscles form the GL-treated rats were significantly higher than that in the controls (0.37 ± 0.05 vs.1.71 ± 0.77, *p* = 0.04, n = 4, Figure 3B and C). These data indicated that treatment with GL extracts enhanced the AMPK activation, which may reduce the body weights and improvement of insulin sensitivity in rats.

## Discussion

In a previous study, we demonstrated that administration of GL extracts may have preventive effects of ameliorating hepatic accumulation and hepatic insulin resistance by enhancing the adiponectin β-oxidation system [26]. However, studies have yet to elucidate the underlying mechanisms of GL extracts on glucose metabolism and insulin resistance in skeletal muscle.

This study was designed to investigate the effects of the ethanol soluble GL extracts on glucose tolerance and insulin sensitivity in the skeletal muscles of SHRSP/ZF rats. We found that oral treatment with GL extracts for six weeks did not alter in the amount of food and water consumption and fasting plasma glucose and insulin levels in SHRSP/ZF rats. However, the treatment did significantly improve glucose tolerance in the rats. Evidentially, the levels of plasma glucose at 60 and 120 min post glucose challenge in the GL-treated rats at 42 days post treatment were significantly lower than that in the control rats. As a result, the values of AUC and QUICKI in the GL-treated rats were significantly less than that in the control rats. These data suggest that GL extracts may enhance glucose challenge-stimulated insulin secretion, which promotes glucose metabolism and improves insulin sensitivity in the major glucose metabolic organs, such as the skeletal muscles, liver, and adipose tissues of SHRSP/ZF rats [27].

Obviously, the skeletal muscle is the main organ responsible for the postprandial hyperglycemia following a meal in normal individuals. Given that previous studies have demonstrated that treatment with GL extracts could stimulate glucose utilization in liver tissues in spontaneous and inducible animal models of diabetes [12-15], our data extended previous findings and indicated that treatment with GL extracts improved glucose tolerance and insulin sensitivity in the skeletal muscles of animals.

Insulin can interact with its receptor and activate IR tyrosine kinase, leading to the activation of IRS-1, PI3K-p85, AKT, and GLUT4 translocation, and promoting glucose uptake and metabolism in the skeletal muscles [4,28]. A defect in the activation of insulin-related signal events is associated with the development of insulin resistance and glucose intolerance [5,25]. To understand the mechanisms underlying the action of GL extracts in regulating glucose metabolism, we characterized the levels of insulin-related signaling in the skeletal muscles. We found that treatment with GL extracts significantly promoted IRS-1, AKT, PI3Kp85 expression and IRS-1, AMKP, and AKT308, but not AKT473, phosphorylation in the skeletal muscles of SHRSP/ZF rats. These data indicated that treatment with GL extracts promoted insulin-related signaling in the skeletal muscles of SHRSP-ZF rats. The enhanced insulin-related signaling should enhance GLUT4 translocation in the muscular cells. Indeed, we found that treatment with GL extracts significantly increased the ratio of membrane to total GLUT4 expression in the skeletal muscles of SHRSP-ZF rats. Conceivably, the increased membrane-related GLUT4 should enhance glucose uptake and accelerate glucose metabolism in the skeletal muscles of SHRSP/ZF rats. Given that insulin resistance is a pathological hallmark of MS and T2DM, the activity of GL extracts in improving insulin sensitivity in the skeletal muscles suggest that GL extracts may be valuable for the control of insulin resistance-related MS and T2DM.

Obesity is associated with the development of insulin resistance and T2DM [29]. In T2DM and obese individuals, insulin-stimulated glucose disposal in skeletal muscle is markedly impaired, because excess fat can deposit and accumulate in the myocytes, impairing insulin sensitivity [30]. The altered fatty acid metabolism in the muscles is also observed in obese Zucker rats and is associated with the development of insulin resistance [31]. In our study, we found that treatment with GL extracts for six weeks significantly reduced the gain of body weight in SHRSP/ZF rats. Given that the loss of body weight in obese individuals can improve insulin sensitivity [32], the effect of GL extracts on significantly reduced body weights may also contributes to its activity in the improvement of insulin resistance in the skeletal muscles of SHRSP/ZF rats. Notably, obesity is a risk factor of the development of MS, T2DM, and hypertension as well as other cardiovascular diseases. Therefore, GL extracts may be beneficial for obese individuals to prevent and treat MS, T2DM, and hypertension.

Adiponectin is an adipokine and insufficient adiponectin has been implicated in the development of obesity and T2DM [8]. AdipoR1 is abundantly expressed in skeletal muscle and at moderate levels in other tissues, whereas AdipoR2 is predominantly expressed in the liver [7]. In previous study, we demonstrated that GL extracts significantly decrease liver triglyceride content of SHRSP/ZF rats via increase the AdipoR1 and AdipoR2 to active the activity of AMPK and PPARα [26]. In present study, although we did not observe that treatment with GL extracts significantly altered the levels of plasma lipids, we found that the treatment significantly up-regulated AdipoR1 expression and increased the AMKP activation in the skeletal muscles of SHRSP-ZF rats. The enhanced AMKP activation by GL extracts increased fatty acid oxidation and decreased fatty acid esterification, inhibiting ACC activity, and suppressing lipogenic enzymes, such as fatty acid synthase and stearoyl CoA desaturase 1 [33], which resulted in the reduction of fat deposition and accumulation in the skeletal muscles. This may contribute to its activity in reducing body weight, ameliorating IRS-PI3K-AKT signaling pathway to improve glucose tolerance and insulin sensitivity in the skeletal muscles. Furthermore, the enhanced AMKP activation may stimulate muscle glucose uptake, this occurs through a distinct mechanism than the insulin-signaling pathway [34].

## Conclusions

In summary, our data indicated that oral treatment with GL extracts reduced the gain of body weight and improved glucose metabolism and insulin sensitivity in SHRSP/ZF rats. Furthermore, we found that treatment with GL extracts significantly enhanced the insulin-related signaling, GLUT4 translocation, AdipoR1 expression, and AMKP activation in the skeletal muscles of SHRSP/ZF rats. It is possible that GL extracts, through activating the insulin signaling and promoting glucose metabolism and fatty acid oxidation, lead to the improvement of insulin sensitivity in the skeletal muscles in SHRSP/ZF rats. Therefore, our findings may provide new insights into the mechanisms by which GL extracts inhibit insulin resistance in the skeletal muscles. Potentially, our findings may aid in the design of new therapies for the prevention and treatment of insulin resistance-related MS, T2DM, and hypertension in the clinic.

## Abbreviations

MS: Metabolic syndrome;T2DM: Type 2 diabetes mellitus;IRS: Insulin receptor substrate;PI3K: Phosphatidylinositol-3-kinase;PKB/AKT: Protein kinase B;GLUT 4: Glucose transporter 4;AMPK: 5′-AMP-activated protein kinase;ACC: Acetyl coenzyme A carboxylase;GL: Guava leaf;PTP1B: Protein tyrosine phosphatase 1B;SHRSP/ZF: SHRSP.Z-Leprfa/Izm;FFA: Free fatty acids;OGTT: Oral glucose tolerance test;AUC: Area under the curve;QUICKI: Quantitative insulin sensitivity check index

## Competing interests

The authors declare no financial or commercial conflicts of interest.

## Authors’ contributions

XG designed the study, conducted the experiments, and wrote the manuscript. YH performed the experiments, analyzed the data. GM designed the study and wrote the manuscript. YD, WS, TX, and PX performed the experiments and analyzed the data and manuscript preparation. LQ and JZ helped to perform the experiment. LH drafted the manuscript. TL provided the initial idea, instructed the study, and wrote the manuscript. All authors read and approved of the final manuscript.

## Pre-publication history

The pre-publication history for this paper can be accessed here:

http://www.biomedcentral.com/1472-6882/13/52/prepub
